# Restless Legs Syndrome, and symptoms of Restless Syndrome in patients with Graves’ disease: a cross-sectional survey

**DOI:** 10.6061/clinics/2020/e2140

**Published:** 2020-11-02

**Authors:** Marcia Pradella-Hallinan, José Carlos Pereira, João Roberto Maciel Martins

**Affiliations:** I Departamento de Pediatria, Faculdade de Medicina de JundiaI, SP, BR; II Departamento de Psicobiologia, Universidade Federal de Sao Paulo (UNIFESP), SP, BR e Hospital Sirio-Libanes, Sao Paulo, SP, BR; III Departamento de Endocrinologia, Universidade Federal Paulista (UNIFESP), Sao Paulo, SP, BR

**Keywords:** Restless Legs Syndrome, Willis-Ekbom Disease, Graves’ Disease

## Abstract

**OBJECTIVES:**

Restless legs syndrome (RLS) is a frequent comorbid condition associated with distinct unrelated diseases. While the incidence of RLS has not been definitively confirmed, RLS-like symptoms have been reported in a section of Asian population who also had hyperthyroidism. The prevalence of RLS is generally low in Asian populations. Under these circumstances, we hypothesized that in a population where RLS is common, such as in Brazil, RLS could manifest as a comorbid ailment alongside Graves’ disease, a common hyperthyroid condition.

**METHODS:**

In a cross-sectional survey, 108 patients who presented with Graves' disease were analyzed for restless legs or associated symptoms.

**RESULTS:**

Twelve patients (11.1%) displayed symptoms of RLS prior to the incidence of Graves’ disease. These patients experienced worsening of the symptoms during their hyperthyroid state. Six patients (5.6%) developed RLS, consequent upon the incidence of Graves’ disease as per the consensus of the panel of the experts. Fifteen patients (13.9%) also presented with RLS-like symptoms without any discernible circadian feature of the syndrome.

**CONCLUSION:**

Our findings confirm that Graves’ disease might trigger restless legs-like symptoms, while the condition of hyperthyroidism could also be complicated by definite RLS.

## INTRODUCTION

Restless legs syndrome (RLS), also known as Willis-Ekbom disease (WED), is a sensorimotor disorder which is diagnosed based on specific clinical criteria (1-4). While the disease occurs mainly in adults, children have also been reportedly affected (3-5). It often manifests as a comorbid condition alongside several unrelated diseases (6). RLS is defined by five cardinal characteristics: a) the patients feel the urge to move their legs when they are either resting or inactive (lying down or sitting); b) their urge is usually accompanied by paresthesia or pain, deep within the legs; c) the symptoms are often relieved by movement; d) the symptoms occur exclusively or predominantly during the evening and night (circadian aspect), and e) the symptoms are not the result of any other medical or behavioral condition (2,3). It is believed that hypofunctioning of the dopaminergic system might underpin the pathophysiology of RLS as dopamine (DA) agonists are very effective in alleviating RLS symptoms (4,7,8).

Among the hypofunctioning dopaminergic systems that could contribute to RLS, the tuberoinfundibular pathway and the diencephalon spinal dopaminergic system are the most promising (4,9-12). In the tuberoinfundibular pathway, one of the four major dopaminergic systems in the brain, dopamine is secreted as a neurohormone in the pituitary stalk. This causes an inhibition of prolactin release and thyroid-stimulating hormone (TSH) (13-15). DA inhibits the secretion of TSH by the pituitary, consequently diminishing TSH-induced actions on the thyroid gland, thereby decreasing thyroid hormone (TH) release. Thus, the excitability of the peripheral somatosensory system also decreases (10,16-22).

It is interesting to note that in the 1970s, researchers observed that intravenous infusion of DA decreased TSH levels (23). Furthermore, pramipexole, one of the best DA agonists used to treat RLS, could decrease TSH levels (24). These two pharmacological facts suggested that an increase in the activity of the thyroid axis might be involved in the genesis of RLS symptoms. Besides, numerous reports indicated the peripheral nervous system as the neuroanatomical site wherein RLS symptoms are initiated (10,).

As mentioned earlier, RLS is associated with various other diseases (6). Thus, it is interesting to investigate the possible presence of RLS or RLS symptoms in patients presenting with a hyperthyroidism state such as Graves' disease (GD). To the best of our knowledge, only three articles examining these effects have been published by Tan EK et al. (28-30) in Singapore. Their findings demonstrated that symptoms of RLS are common in patients with hyperthyroidism. However, they could not find any patient wherein a definite case of RLS could be confirmed as none of the patients displayed any characteristic circadian pattern of RLS such as worsening of symptoms in the evening and night (2). These observations (28,29) indicated that hyperthyroidism did not cause RLS; that is, RLS and hyperthyroidism did not exist as comorbid conditions. However, these investigations were concentrated on patients from Singapore, and the prevalence of RLS in the Asian population is generally very low (only 0.1%) (30). In Brazil, within the state of São Paulo, there is a large population of Portuguese, Italian, and African descendants, and the prevalence of RLS is much higher (occurring in at least 6.4% of the Brazilian adults) (31). These considerations motivated us to conduct this cross-sectional survey to search for RLS symptoms among patients presenting with Graves’ disease.

### Patients and Methods

Over a period of 1 year, we interviewed 108 patients within the age range of 13-71 years with a median age of 46±15.8 years (SD). All patients presented with GD and were outpatients of a clinic for thyroid diseases at the Department of Endocrinology within the Universidade Federal Paulista (UNIFESP), School of Medicine, São Paulo, Brazil. Among the studied population, 65% of the patients were either Portuguese or Italian, approximately 5% were African-Americans, while 30% had mixed ancestry. The patients received treatment for a median of 4.5±4.3 years (SD). All of them were already free of GD symptoms and were in a stable euthyroid condition after treatment with antithyroid drugs and/or radioactive iodine. Although free of GD symptoms, seven patients still presented with laboratory signs of hyperthyroidism. All patients were thoroughly diagnosed as per their laboratory results and clinical diagnosis of GD in the UNIFESP tertiary-care ambulatory unit. We recorded the medical history of only the patients who could clearly recall the RLS symptoms they might have had. As such, our final cohort (after the exclusion of six patients) comprised 108 patients: 94 female (87%) and 14 male (13%). We conducted face-to-face interviews with the patients regarding RLS symptoms they might have had before GD started, during the active phase of GD, and after the hyperthyroidism was cleared. All interviews were administered by one of us (MPH) and the following questions were asked: i) When at rest (lying or sitting), did you feel any odd sensations within your legs during the active period of your GD (for odd sensations, we explained that they were those typical of RLS sensorial symptoms described by the majority of RLS patients); ii) Were these symptoms ameliorated when you moved your legs; iii) Did the symptoms resurface after you stopped your movement; iv) Did you feel the symptoms at least twice a week, even sometimes with an interval of a few weeks without the symptoms; v) Was there any difference in these symptoms according to the time of the day?

Were they more intense in the evening and at night; vi) Did you feel these symptoms before the GD started; vii) If they were present before GD, did they worsen with the thyroid disease; viii) After the thyroid was treated, did the symptoms cease? We did not ask anything about sleep duration or sleep quality of the patients because hyperthyroidism, *per se,* impairs sleep, and this question could be more of a confounder than a clarification. One of us (JCPJr.) also interviewed 36 patients with the same questions within the scope of corroboration of our inquiry. This work was conducted in the Faculdade de Medicina de Jundiaí, and was approved by the Committee of Ethics on Investigative Studies in humans in our institution (2014). 

## RESULTS

The second interviewer (JCPJr.) obtained the same interview responses with the 36 patients as obtained by the first interviewer (MPH), although the emphasis on the responses to each question varied slightly. Based on the interview responses, we were able to obtain four different phenotypes (groups) related to RLS-like symptoms, the results are summarized in Table 1.

Group 1: Patients with GD having RLS symptoms, but without the presence of the circadian characteristic that defines RLS, which is not definite RLS triggered by GD. RLS symptoms were not present prior to GD. There were 15 patients in this group (13.9%). Of them, 11 patients informed us that they did not feel RLS symptoms when they became euthyroid. The other four patients informed that while the symptoms had improved after GD, they were not entirely cured.

Group 2: Patients with GD presenting definite RLS prior to GD. These patients also experienced worsening of RLS symptoms during the hyperthyroid condition. Further, the worsening of the symptoms was more intense in the evenings and nights. There were 12 patients in this group (11.1%).

Group 3: Patients with GD without definite RLS prior to GD. Only after the incidence of GD, definite RLS was manifested. As by its definition, RLS occurred during, and not after, the hyperthyroid condition. There were six patients in this group (5.6%).

Group 4: Patients with GD who did not present any RLS symptoms prior to GD or during GD. There were 75 patients in this group (69.4%).

## DISCUSSION

Our results from the Group 1 phenotype (13.9%) suggest that GD might trigger RLS-like symptoms (mimic RLS), that is, without the typical circadian aspect that characterizes definite RLS. We believe that this might occur because of the high levels of circulating TH in patients with GD, which may be so elevated that the DA systems are unable to counteract the large number of action potentials fired by the hyperexcitability of the somatosensory system, deep inside the legs. We hypothesize that the circadian aspect that defines RLS in normal individuals is a consequence of the circadian increase in TH axis activity in the evening, as mentioned above (20). However, in GD, the thyroid gland is deregulated, and TH levels do not follow the typical circadian rhythm. Instead, the thyroid gland is incessantly stimulated by immunoglobulin directed against TSH receptors that releases TH into circulation. This causes loss of the normal daily TSH levels curve (32). Tan EK et al. observed a similar effect in 8.8% of the patients with hyperthyroid they studied (29). In our view, the difference in percentages between both the surveys stems from the fact that in Brazil, RLS is more common in normal individuals (at least 6.4%) than in Singapore (0.1%) (30). In Group 1, 11 patients informed us that they did not experience RLS-like symptoms when they became euthyroid. The other four patients in Group 1 informed us that the symptoms had improved but were not entirely cured.

In Group 2 of our study, 12 patients (11.1%) presented with RLS prior to GD. This diagnosis was clearly established in face-to-face interviews, although seven patients did not systematically present with typical RLS symptoms twice a week. Seven patients had frequent symptom remission for 2 or more weeks, during which they were free of unpleasant sensations and the urge to move their legs. We observed that 10 patients within this group had light or moderate RLS symptoms as defined by experts in RLS (7). However, only two patients had moderately severe RLS. This prevalence of definite RLS prior to GD (11.1%) is in accordance with the numbers presented in the medical literature (between 5% and 15% in European populations) (2). All 12 patients in this group reported that when GD was initiated, they experienced the worsening of RLS symptoms that were present before GD. Symptoms occurred much more frequently, paresthesia or pain was more intense, and the urge to move their legs increased. The typical RLS symptoms of the patients in Group 2 continued to follow the circadian characteristic of RLS, which involved worsening at night. As such, given that the levels of TH in GD are continuous during the day (32), we believe that a decline in the DA counteracting systems during the night contributes to the continuity of the circadian characteristic of definite RLS in these patients. The results from the patients in Group 2 patients suggest that not only does the thyroid axis activity need to increase at night for the fourth criterion of RLS to manifest; but also a larger than normal decline in DA counteracting systems might evince it. Of the 12 patients in Group 2, six informed us that their definite RLS returned to baseline when they were not experiencing GD; four patients’ RLS had ameliorated after GD treatment, and the other two patients informed us that they were not sure if their RLS had been ameliorated after GD treatment.

Among the 108 patients with GD, six (Group 3), presented with definite RLS after GD, but they did not have any RLS symptoms prior to GD, suggesting that in some patients, hyperthyroidism might lead to the development of definite RLS as a comorbidity. As explained above, during GD, TH levels do not have any specific circadian characteristics (32). Notwithstanding, 5.6% of our patients with GD developed all the characteristics typical of definite RLS. We hypothesize that the neurohormone DA, in its daily curve of liberation, decreases during the evening and night more than normal, making it unable to counteract the hyper-excitability of the peripheral sensitive nerves deep inside the legs (4), because of an increase in the circulating TH levels in the patients with GD in Group 3.

Seventy-five (69%) of our patients (Group 4) did not present with either definite RLS or symptoms that mimicked RLS. This suggests that these patients were not prone to develop RLS or that the systems they had to counteract elevated TH levels were effective in their regulatory functions in peripheral somatosensory system activity.

## CONCLUSIONS

The results obtained from this cross-sectional survey suggest that hyperthyroidism might trigger RLS-like symptoms or even definite RLS, as specified by a committee of experts on this disease. Pramipexole, a first-line therapeutic drug against RLS, diminishes the circulating levels of TH, which is considered a side effect of this dopaminergic agonist. It is possible that this “side effect” is indeed a therapeutic action of pramipexole against RLS/WED.

This study was approved by the ‘‘Committee of Ethics of Research on Humans’’ of our institution, Faculdade de Medicina de Jundiaí, São Paulo, Brazil.

## AUTHOR CONTRIBUTIONS

Pradella-Hallinan M conceived and designed the study, conducted the face-to-face patient interviews and contributed toward writing the manuscript and researching pertinent literature. Pereira Jr JC contributed to the face-to-face interviews, writing of the manuscript as well as researching pertinent literature. Martins J contributed with his expertise on Graves’ disease in the studied patients.

## Figures and Tables

**Table 1:** Phenotypes in relation to RLS-like symptoms obtained in this inquiry.

NUMBER OF PATIENTS	PHENOTYPES
GROUP 1 15 PATIENTS(13.9%)	Patients without RLS prior to GD and those who developed RLS-like symptoms, without the definite RLS circadian characteristics, during GD
GROUP 212 PATIENTS(11.1%)	Patients presenting definite RLS prior to GD who also experienced worsening of RLS symptoms during GD
GROUP 36 PATIENTS(5.6%)	Patients without definite RLS prior to GD and who developed definite RLS during GD
GROUP 475 PATIENTS(69.4%)	Patients with GD who did not develop any symptom of RLS
